# Consumption in out-of-hours health care: Danes double Dutch?

**DOI:** 10.3109/02813432.2014.898974

**Published:** 2014-03

**Authors:** Linda Huibers, Grete Moth, Mikkel Andersen, Pierre van Grunsven, Paul Giesen, Morten Bondo Christensen, Frede Olesen

**Affiliations:** ^1^Research Unit for General Practice, Department of Public Health, Aarhus University, Denmark; ^2^Scientific Institute for Quality of Healthcare, Radboud University Medical Center, The Netherlands; ^3^Research Department Prehospital Emergency Medical Services, Aarhus, Denmark, and Department of Clinical Epidemiology, Aarhus University Hospital, Denmark; ^4^Emergency Medical Service Gelderland-Zuid, the Netherlands

**Keywords:** After-hours care, Denmark, health services research, primary care, the Netherlands, utilization

## Abstract

*Objective.* To study the quantitative consumption in out-of-hours (OOH) primary care in Denmark and the Netherlands, in the context of OOH care services. *Design.* A retrospective observational study describing contacts with OOH care services, using registration data. *Setting.* OOH care services (i.e. OOH primary care, emergency department, and ambulance care) in one Danish and one Dutch region. *Subjects.* All patients contacting the OOH care services in September and October 2011. *Main outcome measures.* Consumption as number of contacts per 1000 inhabitants in total and per age group per contact type. *Results.* For the two-month period the Danes had 80/1000 contacts with OOH primary care compared with 50/1000 for the Dutch. The number of contacts per 1000 inhabitants per age group varied between the regions, with the largest difference in the 0–5 years age group and a considerable difference in the young-adult groups (20–35 years). The difference was largest for telephone consultations (47/1000 vs. 20/1000), particularly in the youngest age group (154/1000 vs. 39/1000). The Danes also had more home visits than the Dutch (10/1000 vs. 5/1000), while the Dutch had slightly more clinic consultations per 1000 inhabitants than the Danes (25/1000 vs. 23/1000). *Conclusion.* The Danish population has more contacts with OOH primary care, particularly telephone consultations, especially concerning young patients. Future research should focus on the relevance of contacts and identification of factors related to consumption in OOH primary care.

- Danish and Dutch health care systems are quite comparable, having strong primary care and large-scale out-of-hours (OOH) primary care settings.
- National figures suggested that the Danish population has twice as many contacts with OOH primary care as the Dutch.
- A regional exploration confirmed this: the Danes generally had more OOH primary care contacts than the Dutch, particularly telephone consultations.
- The difference is most evident for the youngest patients, which may be explained by differences in organizational and patient-related factors.

## Introduction

In many countries, the organization of out-of-hours (OOH) primary care has been changing, primarily because of dissatisfaction among general practitioners (GPs), shortage of GPs, and high workload [1–5]. OOH primary care patient demands are increasing, mainly due to non-urgent contacts [6]. Because of high demands and extensive opening hours, many patients have a health care contact with OOH care.

Denmark and the Netherlands are quite comparable, being welfare states with similar health care systems, including a strong general practice acting as gatekeeper (Table I) [7–10]. In general, Danish and Dutch patients have three options to contact health care whenever they experience an acute health problem: general practice, emergency departments (EDs), and ambulance care services. Outside office hours, primary care is organized in large-scale settings, with telephone triage to manage patient flows. Yet, different triage professionals are used, with GP telephone triage in Denmark and physician assistant/nurse triage – supervised by GPs – in the Netherlands [2,4,11,12].

**Table I.** Characteristics of Danish and Dutch primary care.

Characteristics	Denmark	The Netherlands
Daytime primary care:		
Number of patients per full-time GP	1600	2250 to 2500
Opening hours	8 a.m. to 4 p.m.	8 a.m. to 5 p.m.
Practice staff	About 0.7 staff per 1 full-time GP	About 1.3 staff per 1 full-time GP
Role in palliative care	Own GP takes care of patients staying at home	Own GP takes care of patients staying at home
Care during pregnancy	By GPs and midwives	Mostly by midwives
Preventive care children	By GP	Special organizations for vaccination and prevention, using specialist physicians; cure by GPs
Surgical procedures	Standard minor surgery (extra fee-for-service)	Standard traumata and elective surgery (extra fee-for-service 63 euros)
Home care	Average home visits by own GP 3 per week; also special home care nurses	Special home care nurses; home visits by own GP average of 20 per week
Threshold to primary care	No additional payment	No additional payment
Payment of GPs	Capitation 30% and fee-for-service 70%	Capitation 70% and fee-for-service 30%
Out-of-hours acute care:		
Number of GPs per setting	Per setting about 300 to 1000 GPs are linked, but not all do the OOH shifts themselves (in some regions less than 50%)	50 to 250 GPs per setting
Number of patients per setting	580 000 to 1 736 000	100 000 to 500 000
Opening hours	4 p.m. to 8 a.m., weekends, holidays	5 p.m. to 8 a.m., weekends, holidays
Distance to consultation centre for OOH primary care	Distance is max 50 km	Distance on average is max 30 km
Telephone triage	GPs	Nurses and physician assistants, supervised by GPs
Role in palliative care	Both GPs themselves and outsourced to the OOH primary care	75% themselves or colleagues in daily practice, 25% outsourced to the OOH primary care
Role ED–OOH primary care–ambulance	Shared telephone access in one region	Increasing tendency of collocation and collaboration of primary care setting and ED (about 70% collaborating)
Threshold to care to acute care	No additional payment	No additional payment
Financing of OOH primary care	OOH primary care is fully covered by the nationwide tax-paid health insurance (public funding), expect for medication Citizens can opt for private insurance additionally	The statutory health insurance system is financed through a nationally defined income-related contribution and through community-rated premiums set by each insurer (private insurance) OOH primary care is fully covered by the insurance, except for medication and diagnostic tests (own risk for citizen)
Payment of GPs	Fee-for-service, varying for type of contact	Fee per hour (63 euros)
Re-registration as GP	No criteria	A minimum of 50 hours of OOH shifts per year is a criterion for re-registration as a GP (25 hours for GPs with a GP registration > 25 years)

Note: For further detailed information about the Danish and Dutch health care systems see overview articles [7–10,21,22]. ED = emergency department; OOH = out-of-hours.

Interestingly, patients’ use of OOH primary care seems to differ considerably between the countries, with 249 contacts per 1000 patients per year in the Netherlands compared with 503 contacts in Denmark [13,14]. It seems unlikely that Danish citizens have more medical health problems and, therefore, other patient and organizational-related factors have to contribute to this difference. The Dutch may have more contacts with other OOH health care providers or with their own GP, resulting in fewer contacts with OOH primary care. Furthermore, due to the GP triage, Danish patients have direct access to a fully trained GP OOH [4,9], which could trigger patients to contact. Besides, Danish and Dutch citizens may differ in their threshold for seeking help OOH.

Studying the difference in consumption of OOH primary care is relevant, as insight into reasons for differences could provide input regarding how to plan appropriate use of health services. A study of two countries with comparable health care has the ability to go beyond national boundaries of the organization of OOH primary care and study different models. Thus, our aim was to study the quantitative consumption in OOH primary care in Denmark and the Netherlands, to investigate the expected difference, and to explore the distribution of contact types, in the light of different organizations in the triage process.

## Material and methods

### Design and settings

We performed a retrospective observational study to describe contacts with OOH care services in one Danish and one Dutch region, using registration data from the regional OOH care settings. We focused on OOH primary care settings, but we also included the other main OOH care settings (i.e. EDs and ambulance care service) and we collected information on the consumption in daytime GP practices.

In Denmark, health care is organized within five well-defined regions and background information on the region is available online [14,15]. In the Netherlands, regional health care settings do not have identical catchment areas, so we defined the Dutch region as the catchment area of the OOH primary care setting. The Danish region was the Central Denmark Region, containing one OOH primary care setting, 11 EDs, and one ambulance care service (see Supplementary Appendix 1 available online at http://informahealthcare.com/doi/abs/10.3109/02813432.2014.898974). The Dutch region was located in the south-east of the Netherlands, having one OOH primary care setting, three EDs, and two ambulance care services. The Danish OOH primary care service is open from 4 p.m. to 8 a.m. on weekdays, at weekends, and on public holidays, while the Dutch service starts at 5 p.m. (see Table I for more details).

### Data collection in OOH primary care

We collected anonymous data sets from the registration systems of the OOH primary care settings, containing all contacts for September and October 2011. We collected the following variables for all contacts: patients’ age and gender, date, time, and type of contact (i.e. telephone consultation, clinic consultation, and home visit). Information on population characteristics was obtained from the website of Statistics Netherlands [16].

### Additional data collection consumption

We also aimed to get data for all contacts with the other OOH care settings in the same period. For the Danish region we received data for all contacts with the EDs and ambulance care. Concerning the Dutch EDs and ambulance care service we selected contacts from (a part of) the OOH primary care catchment area using zip codes. Regarding the contacts with GPs during office hours, the Danish number was extracted from a national registration system, including only billed contacts. The Dutch number was estimated, based on the national figure of the Netherlands Information Network of General Practice (LINH) [17].

### Analysis

We performed descriptive analyses, using chi-squared tests. Consumption was presented as the average number of contacts per 1000 inhabitants for the two months, using the information on population characteristics from Statistics Denmark and Netherlands Statistics [14,16]. We also calculated the number of contacts per age group per 1000 inhabitants per contact type, thereby correcting for differences in age distribution. Furthermore, we performed a sensitivity analysis to study the effect of the longer opening hours in Denmark. We added the highest number of contacts per hour to the total number of contacts for the Dutch region, recalculating the rate per 1000 inhabitants.

## Results

### Population characteristics

The populations of Denmark and the Netherlands were quite comparable on a national and regional level concerning age and gender, although the youngest age group was larger in the Danish region (Table II).

**Table II.** Population characteristics: National and regional (n and %).

	Denmark		The Netherlands	
	National 2011	Regional 2011	National 2011	Regional 2011
Inhabitants (n)	5 560 628^1^	1 265 601^1^	16 655 799^2^	430 498^3^
Males (%)	49.6	49.9	49.5	49.2
Age in years (%):
0–20	24.3	25.1	23.5	22.8
20–40	24.8	25.2	25.0	25.3
40–65	34.1	33.4	35.9	36.8
65–80	12.7	12.4	11.6	11.6
80 +	4.1	3.9	4.0	3.6

Notes: ^1^Statistics Denmark, 2011M1 and 2011K4; ^2^Statistics Netherlands, 2011M1; ^3^Number of inhabitants was calculated with data from Statistics Netherlands, 2011M1, using the zip codes of the service area of the Dutch OOH primary care setting; due to anonymity protection numbers with age groups were rounded off.

### Regional contacts with OOH primary care

The percentage of male patients was almost identical in Danish and Dutch OOH primary care (44.8% and 45.6% respectively), but the age distribution varied, the Danish patients being younger than the Dutch (average age 33.9 versus 41.3 years; Table III). Furthermore, the distribution of contact types varied between the Danish and Dutch regions, particularly concerning telephone consultations (58.6% versus 40.3%) and clinic consultations (28.4% versus 50.2%).

**Table III.** Contacts with out-of-hours primary care in the Danish and Dutch region for September and October 2011 (n and n/1000).

	Denmark^1^ (inhabitants ≈ 1 265 601)				The Netherlands^2^ (inhabitants ≈ 430 498)			
	Telephone n = 59 378	Clinic n = 28 790	Visit n = 3261	Total n = 101 429	Telephone n = 8619	Clinic n = 10 611	Visit n = 2180	Total n = 21 410
Contacts (%)	58.6	28.4	13.1	100	40.3	49.6	10.2	100
Gender (% male)	42.8	48.2	46.7	44.8	40.0	50.2	45.0	45.6
Age (mean)	32.4	26.6	56.9	33.9	43.4	33.5	70.8	41.3
Contact rate (n/1000)	47	23	10	80	20	25	5	50

Note: ^1^Data from the National Health Insurance Service Registry; ^2^Registration data OOH primary care service.

**Figure 1.**
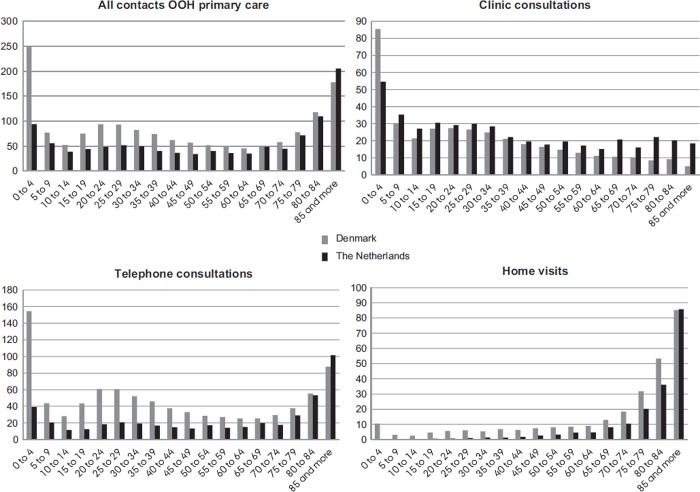
Number of contacts per 1000 inhabitants per age group Denmark and the Netherlands (for all contacts and per contact type). Note: *Most differences were significant (p < 0.05).

For the two-month period, the overall number of contacts was 80 per 1000 inhabitants in the Danish region, compared with 50/1000 in the Dutch region (see Table III). The largest differences were for the age groups 0–5 years and 20–35 years (Figure 1). In the age group 85 and older, the Dutch had more contacts. This difference in consumption was significant for all three types of contact, but was most profound for telephone consultations (47/1000 versus 20/1000), particularly for the youngest age group (154/1000 versus 39/1000). The Danish region had more home visits than the Dutch (10/1000 versus 5/1000); this difference slightly decreased with increasing age. The Dutch had slightly more clinic consultations than the Danes (25/1000 versus 23/1000), except for the youngest age group and the young adult group.

### Regional frequency of contacts

The figures on regional consumption did not show evidence that contacts with other settings could explain the difference in contacts with OOH primary care ( Supplementary Appendix 2 available online at http://informahealthcare.com/doi/abs/10.3109/02813432.2014.898974). The number of contacts with daytime primary care was 1146 per 1000 inhabitants in the Danish region, while an extrapolation of the Dutch national figure gave 717/1000. The contact frequency with the ED and ambulance care was 25/1000 and 20–21/1000 in the Danish region versus 15–24/1000 and 10–20/1000 in the Dutch region.

A sensitivity analysis was performed to correct for the difference in opening hours, using raw data from the Danish OOH primary care. This resulted in a minimal increase in contacts (3 per 1000) for the Dutch region (data not shown).

## Discussion

### Statement of principal findings

The Danish population had almost twice as many contacts with OOH primary care compared with the Dutch population. This difference did not seem to be associated with an increased number of contacts with other care settings by the Dutch. In general, the share of young patients contacting OOH primary care seemed to be higher in Denmark. This difference was most apparent for telephone consultations. Furthermore, the Danes had more home visits and fewer clinic consultations than the Dutch.

### Strengths and weaknesses of the study

We were able to give a general overview of regional health care consumption, using considerable data sets and presenting the number of contacts per 1000 inhabitants. Thus, our results give a realistic overview of consumption, with input for future studies. Yet, the studied countries used varying registration systems and had some disparities in registration, related to the payment system, particularly for telephone consultations. Therefore, we cannot completely eliminate bias. However, estimated figures showed that the influence of the registration disparities on the displayed difference is likely to be limited.

Some specific limitations existed regarding the data collection. The provision of Dutch OOH care is not organized within predefined regions, so we had to define a region. Consequently, we chose to present a range of contacts for the ED and ambulance care instead of a point estimate, to take the uncertainty into account. Using information from Statistics Netherlands, we were able to study consumption and calculate contact rates.

### Findings in relation to other studies and interpretation

Previous national figures also showed a considerable difference in consumption between Danish and Dutch OOH primary care [13,14]. One hypothesis could be a shift of Dutch patients to other health care settings, but our figures did not support this. We cannot evaluate the difference found as Danish over-consumption or Dutch under-consumption, so we reflect on possible reasons for the difference.

The difference in consumption was largest for the youngest age group (0–5 years), particularly for telephone consultations. This may be related to the high number of non-urgent contacts and the large share of contacts for young children in OOH primary care, while triage GPs more frequently end a contact by telephone [13,14,19,20]. Furthermore, Danish patients (parents) may have a lower threshold for seeking help and a different assessment of severity of health problems. Parents being aware of the direct access to a GP OOH could also contribute to the higher contact frequency, for example if they need formal GP advice concerning the child being able to attend day care the next day, a situation which may be more typical for Danish women, who more often have a full-time job than Dutch women [18]. Also, Danish GPs have a profound role in counselling and preventive care for children, as well as pregnancy care and care for the elderly, while other health professionals often perform these tasks in the Netherlands. Danish patients may therefore be more used to contacting their GP for a broad range of health problems, which may influence patient behaviour and lower the threshold to contact GPs OOH [10].

Yet, the higher contact frequency in the Danish region could represent the strengths of an optimal welfare state with easy 24-hour access to professional advice from a GP in the front line of the OOH care, providing a good service. The higher number of home visits could also be service-related, although is probably also related to geographic differences. The slightly higher frequency of clinic consultations in the Dutch region may be related to nurse telephone triage, with a lower threshold for face-to-face contacts.

It seems highly unlikely that Danish citizens have more health problems needing acute care, considering the comparable economic and social status of the countries [18]. Organizational factors could affect the consumption rate, such as differences in opening hours, the payment system, and access to GPs (see Table I ) [7–10]. The extra opening hour gives the Danes more time to pursue an OOH contact. However, the OOH primary care settings have identical contact patterns with a peak directly after opening hours (personal communication) [20] and our sensitivity analysis showed that the extra hour did not influence the difference in consumption greatly. Also, the fee-for-service system in Denmark potentially influences registration and GP behaviour [8,10]. Danish triage GPs receive a higher fee if they handle the contact by telephone than if they refer for a face-to-face consultation, based on the assumption that telephone consultations are generally more time consuming [4]. However, one could argue that, with a fee-for-service, GPs might accept a lower threshold for contacting and refrain from educating patients to call only if necessary. Also, the direct telephone access to a fully trained GP OOH may induce more OOH contacts, especially if patients perceived a limited accessibility to their own GP.

### Meaning of the study: Implications

Further research is warranted to identify all factors relevant to the difference in consumption and the effects on quality of care, ultimately giving input for interventions. Research should focus on patient-related and organizational factors, which could be associated with the difference found. In the future, focus on information concerning self-care and use of OOH services could be part of an intervention, while maintaining quality of care.

## Conclusion

Our exploratory study showed that the Danish population has more contacts with OOH primary care, mainly due to more telephone contacts. In particular, the number of contacts with young patients is higher. Although there are differences in registration of contacts, it is unlikely that these can account for the difference in consumption, which should be addressed in future research and intervention strategies to modify use of OOH services.
