# Supplementary material for: Consumption in out-of-hours health care: Danes double Dutch?
Source: Scand J Prim Health Care. 2014 Mar;32(1):44–50. doi: 10.3109/02813432.2014.898974 (PMC4137903; doi:10.3109/02813432.2014.898974)
Supplement: Supplementary file 1 [file pri-32-44-s001.pdf]

Supplementary material for Huibers L, et al. Consumption in out-of-hours health care: Danes double Dutch? Scand J Prim Health Care 2014;32:44–50.

Supplementary Appendix 1. Description of the OOH care settings in the Danish and Dutch regions.

	Danish region	Dutch region
Characteristics of region		
Size	13 124 km^2 (≈30% of Denmark) and ≈1 265 601 inhabitants	Estimate 842 km^2 and ≈430 498 inhabitants
Urban and rural	One big city, smaller cities, and small villages	One big city, smaller cities, and small villages
Existing health care services		
Primary care[1,2]	Organization: large OOH primary care setting<br>Opening hours: between 4 p.m. and 8 a.m. on weekdays, during weekends, and during holidays<br>Access: a telephone contact is mandatory to get a consultation<br>Telephone triage: by GPs<br>Locations: 13 consultation centres (nine permanent consultation centres located at hospitals and four flexible consultation rooms)<br>Other: on two small islands, local GPs provide acute care	Organization: large OOH primary care setting, collocated with an ED<br>Opening hours: between 5 p.m. and 8 a.m., during weekends, and during holidays<br>Access: in principal a telephone call is mandatory, but patients can walk in; also walk-in ED patients are redirected to primary care<br>Telephone triage: physician assistants and nurses supervised by a telephone GP<br>Locations: one main consultation centre, including call centre and a second consultation centre
ED[1,3,4]	Locations: 11 EDs/acute clinics (five in western part; six in eastern part)<br>Access: in principle patients have to call first (in the western part the general practice; in the eastern part a special telephone number).	Locations: three EDs (two general hospital and one academic hospital); two are collocated with the primary care setting, sharing one entrance<br>Access: in principle persons have to call first
Ambulance /112[1,5]	Organization: ambulance service and communication centre for ambulance care<br>Access: national 112 centre refers medical calls to regional emergency medical communication centre (EMCC)	Organization: ambulance service and communication centre for ambulance care (communication centre shared with fire department and police)<br>Access: national 112 centre refers medical calls to regional emergency medical communication centre
	Telephone triage: nurses/ambulance professionals answer calls, estimating urgency and health care required. GPs and hospitals contact the EMCC directly, bypassing the 112 system	Telephone triage: nurses answer calls, estimating urgency and health care required, and coordinate ambulance transport<br>Addition: nurses handle calls for other health care professionals OOH (e.g. community health services, forensic physicians, maternity care, youth care)

Notes: ED = emergency department; OOH = out-of-hours.

Supplementary Appendix 2. Regional contacts with health care in September and October 2011.

	Danish region (n ≈ 1 265 601 inhabitants)		Dutch region (n ≈ 430 498 inhabitants)	
Service	n (%)	n/1000/2 months	n (%)	n/1000/2 months
GP daytime	1 450 576	1146		717[1]
GP out-of-hours care	101 429	80	21 410	50
Telephone consultation	59 378 (58.6)	47	8619 (40.3)	20
Clinic consultation	28 790 (28.4)	23	10 611 (49.6)[2]	25
Home visit	13,261 (13.1)	10	2180 (10.2)	5
Emergency department	31 536	25	3164 + 4376[3]	5–10 + 10–14
Ambulance dispatch centre[4]	24 803[5]	20–21	3152[6]	10–20

Notes: *In both countries, the numbers presented include some duplicates, due to repetitive contacts by patients or referrals by GPs. [1]National LINH (Netherlands Information Network of General Practice) figure adjusted to represent a two-month period; [2]Walk-in patients visit primary care first, due to collocation with ED and shared entrance; [3]Academic + regional hospital; including range for variation in catchment area; [4]Presenting range, with and without patient telephone calls without dispatch (category E); 6% in Denmark and estimated as up to 50% in the Netherlands; [5]Prospective data collection at ambulance dispatch centre; [6]Ambulance region with number of inhabitants adjusted to safety region.
